# Monitoring influenza vaccination coverage among older adults: a rural cohort study, Rio Grande, 2017-2022

**DOI:** 10.1590/S2237-96222026v35e20250561.en

**Published:** 2026-03-23

**Authors:** Ana Claudia Klein de Almeida de Chaves, Rodrigo Dalke Meucci

**Affiliations:** 1 Universidade Federal do Rio Grande, Saúde Pública, Rio Grande, RS, Brazil; 2 Universidade Federal do Rio Grande, Faculdade de Medicina, Programa de Pós Graduação em Saúde Pública, Rio Grande, RS, Brazil

**Keywords:** Vaccination Coverage, Influenza Vaccines, Elderly, Rural Areas, Rural Health, Cobertura de Vacunación, Vacunas contra la Influenza, Ancianos, Medio Rural, Salud Rural

## Abstract

**Objectives:**

To estimate influenza vaccination coverage in older adults from the municipality of Rio Grande rural cohort, Rio Grande do Sul state, Brazil, using baseline data from 2017 and follow-up data from 2018-2019 and 2020-2022, and to identify factors associated with vaccination uptake during these periods.

**Methods:**

This was a cohort study, based on part of the EpiRural Rio Grande project, conducted at three points in time: baseline in 2017 and follow-up in 2018-2019 and 2020-2022. A total of 651 participants aged 60 years or older were included in all waves. Vaccination information was self-reported. Vaccination coverage proportions (%) were estimated at each follow-up point. Analysis of factors associated with vaccination used hierarchical ordinal logistic regression to estimate odds ratios (OR) with 95% confidence intervals (95%CI).

**Results:**

Influenza vaccination coverage increased from 71.5% at baseline to 85.7% in 2020-2022. In the adjusted analysis, the highest odds of vaccination was associated with not working (OR 1.93; 95%CI 1.24; 2.99), not smoking (OR 2.44; 95%CI 1.45; 4.12), being a former smoker (OR 2.71; 95% CI 1.61; 4.59), diagnosis of pulmonary emphysema (OR 1.85; 95%CI 1.04; 3.28), using long-term medication (OR 1.62; 95%CI 1.06; 2.40), and seeking care at a primary health care center in the last year (OR 2.55; 95%CI 1.82; 3.63).

**Conclusion:**

Influenza vaccination coverage increased over time, although below the national target, and was higher among participants linked to health services and with chronic conditions.

## Resumen

**Objetivos:**

Estimar la cobertura de vacunación contra la influenza en adultos mayores de la cohorte rural de Rio Grande, en estado de Rio Grande do Sul, Brasil, utilizando datos basales de 2017 y datos de seguimiento de 2018-2019 y 2020-2022, e identificar los factores asociados con la trayectoria de vacunación durante estos periodos.

**Métodos:**

Estudio de cohorte, parte del proyecto EpiRural Rio Grande, realizado en tres momentos: basal en 2017 y de seguimiento en 2018-2019 y 2020-2022. Se incluyeron 651 participantes de 60 años o más en todas las fases. La información sobre vacunación fue autodeclarada. Se estimaron las proporciones de cobertura de vacunación (%) en cada punto de seguimiento. El análisis de los factores asociados con la vacunación utilizó regresión logística ordinal jerárquica para estimar las razones de momios (*odds ratio*, OR) con intervalos de confianza del 95% (IC95%).

**Resultados:**

La cobertura de vacunación contra la influenza aumentó del 71,5% al inicio del estudio al 85,7% en el período 2020-2022. En el análisis ajustado, la mayor probabilidad de vacunación se asoció con no estar empleado (OR 1,93; IC95 %: 1,24; 2,99), no fumar (OR 2,44; IC95 %: 1,45; 4,12), ser exfumador (OR 2,71; IC95 %: 1,61; 4,59), diagnóstico de enfisema pulmonar (OR 1,85; IC95 %: 1,04; 3,28), usar medicación continua (OR 1,62; IC95 %: 1,06; 2,40) y haber buscado atención en una unidad de atención primaria de salud en el último año (OR 2,55; IC95 %: 1,82; 3,63).

**Conclusión:**

La cobertura de vacunación contra la influenza aumentó con el tiempo, aunque por debajo del objetivo nacional, y fue mayor entre los participantes con vínculo con servicios de salud y con enfermedades crónicas.

**Ethical aspects**

This research respected ethical principles, having obtained the following approval data:

Research ethics committee: Universidade Federal do Rio Grande

Opinion number: 15/4/2018

Approval date: 2/8/2018

Certificate of submission for ethical appraisal: 70294317.0.0000.5324

Informed consent form: Obtained from all participants prior to data collection.

## Introduction 

Since 1955, the global population aged 60 or over has increased sixfold, and it is estimated that this group will become the majority in the coming decades (1). In Brazil, elderly people are defined as those aged 60 or over (2). Between the 2010 and 2022 Demographic Censuses, the elderly population grew by 56%, and Rio Grande do Sul was the state with the highest proportion, 20% (3).

Population aging is associated with an increase in chronic health conditions and greater vulnerability to infectious diseases, such as respiratory illnesses. It is estimated that approximately 1 billion influenza cases occur worldwide each year, of which 3 to 5 million develop into severe forms, resulting in up to 650,000 deaths (4). In Brazil, in 2024, elderly people accounted for 40% of hospitalizations for severe acute respiratory syndrome caused by influenza, with a 66% case fatality ratio (5).

Influenza vaccination is one of the main public health strategies for reducing severe cases, hospitalizations and deaths, especially among older people. In Brazil, vaccination has been offered annually by the country’s Unified Health System since 1999, with older people being in the priority group (6).

However, influenza vaccination coverage among rural elderly people has not been explored in national and international research, so few studies address this population (7,8). It is estimated that elderly people living in rural areas face greater difficulties in accessing health services, due to distances, lower income and a focus on curative care, which can increase vulnerability and impair quality of life (9).

Since 2017, the national annual target for influenza vaccination coverage has been 90% (10). Although coverage reached this target in previous years, its evaluation at different times is essential to understand the evolution of vaccination among older people, especially in rural areas, where there are barriers to access to health services. The period 2020-2022, marked by the COVID-19 pandemic, represents a unique context, with possible impacts on both the demand for and supply of the vaccine.

From 2021 onwards, vaccination coverage fell below the national target (11), even considering the updated denominator used to calculate vaccination coverage among older people. Even so, the total number of doses administered was 4 million less than in 2020. In 2022, 2023 and 2024, coverage also remained below expectations (12). This heightens the importance of studies that investigate the evolution of coverage in previous periods and identify factors associated with vaccination, which contributes to monitoring trends and planning public health strategies.

The objectives of this study were to estimate influenza vaccination coverage among older adults from the rural cohort of the municipality of Rio Grande, in the state of Rio Grande do Sul, based on baseline data from 2017 and follow-up data from 2018-2019 and 2020-2022, and to identify factors associated with vaccination uptake during these periods.

## Methods 

### Design 

This is a study is based on part of the research entitled “EpiRural Rio Grande: cohort of elderly people from the rural area of the municipality of Rio Grande, Rio Grande do Sul state”, the methodological design of which has been described previously (13). The overall objective of the cohort is to describe and monitor the patterns of morbidity and mortality and use of health services among elderly people residing in the rural area of ​​Rio Grande.

This is a prospective cohort study involving follow-up of this population at three distinct points in time. The baseline was established in 2017, with two follow-up waves: the first between 2018 and 2019 and the second between 2020 and 2022 – the latter was divided into two periods due to the consequences of the COVID-19 pandemic (November 3, 2020–November 26, 2020 and October 4, 2021–January 19, 2022). Our analyses took into consideration the 651 participants present at all three points in time.

### Setting 

This study was developed in the rural area of ​​the municipality of Rio Grande, located in Rio Grande do Sul, the southernmost state in Brazil.

At the time of the study, the municipality had approximately 191,000 inhabitants and, in 2022, 8,383 people resided in rural areas, accounting for 4.4% of the total population (14). The municipality’s rural area had a large territorial extension and low population density, which made access to health services difficult.

The baseline occurred in 2017 and the follow-ups in 2018-2019 and in 2020-2022, which allowed for evaluation of vaccination at different times, including during the COVID-19 pandemic.

### Participants 

Elderly people aged 60 or older, as defined by the Statute of the Elderly (2), residing in the rural area of ​​Rio Grande were included in the study. Those hospitalized or institutionalized during the period when the questionnaires were administered were excluded.

The sample was population-based and was obtained from a household survey conducted in the rural area of ​​the municipality. Participant follow-up was carried out through home visits at three data collection points (2017, 2018-2019, and 2020-2022).

For the longitudinal analyses, all individuals present at all three stages of the research were considered, totaling 651 participants.

### Variables 

The outcome for estimating influenza vaccination coverage at different times focused on the answer to the question: “Have you received any doses of this vaccine since <month> last year?”. The answer options were “yes” and “no”. This question made it possible to assess annual vaccination coverage, as recommended by the Ministry of Health (11). The question was repeated in the same way at all three points in time.

In order to assess the factors associated with influenza vaccination uptake, an ordinal variable was built, derived from the sum of affirmative answers to the question about influenza vaccination in the three periods evaluated (baseline, first wave of follow-up, and second wave of follow-up). The resulting variable had four categories: 0, 1, 2 and 3 vaccine doses received over time, which was interpreted as an indicator of influenza vaccination uptake during the study period.

The exposure variables were grouped into sociodemographic categories (age group, sex, labor activity and living alone). The behavioral characteristics measured included tobacco smoking and alcohol consumption in the last week. The question regarding tobacco smoking had three answer options: never smoked, smoker and former smoker.

Variables related to health conditions included diagnoses of hypertension, diabetes, pulmonary emphysema and asthma. In addition, variables related to long-term medication use and care at a primary health center in the last year were considered.

These variables were considered potential factors associated with vaccination were and included in the hierarchical model as possible predictors and confounders.

### Data sources and measurement 

The data used in this study came from the EpiRural cohort. At all data collection points (baseline, first and second follow-ups), a standardized and pre-coded questionnaire was used, administered by trained interviewers during home visits, with data recorded on tablets using the Research Electronic Data Capture program.

Information on vaccination, sociodemographic characteristics, behavioral characteristics, health conditions and use of health services was obtained by means of participant self-reporting and, when not possible, by their caregiver.

The same data collection instruments and procedures were adopted in all periods, which ensured comparability between the different points in the study.

### Bias 

In order to minimize information bias, a standardized questionnaire was used, administered by trained interviewers, with the same question about vaccination repeated at all three data collection points.

The possibility of attrition bias due to loss to follow-up was acknowledged. To reduce this effect, the analysis was restricted to the 651 participants present in all waves. Potential confounders were controlled using hierarchical multivariate regression.

### Study size 

When the study baseline was performed in 2017, 2,218 households were sampled, accounting for approximately 80% of the total in the rural area of ​​Rio Grande. Selection took place by means of systematic random sampling in all census tracts, with inclusion of four out of every five consecutive households. In these households, 1,130 people aged 60 or over were identified, of whom 1,029 participated in the survey, constituting the initial sample of the cohort (13).

At first follow-up, in 2018-2019, 862 elderly people were re-interviewed (follow-up rate: 83.8%). At second follow-up, in 2020-2022, 651 participated (63.3% of the original sample). For the longitudinal analyses, only the 651 individuals present at all three points in time were included, in order to ensure comparability between the periods.

A power analysis was performed considering the final sample of 651 individuals. The sample size conferred a minimum statistical power of 80%, with a 95% confidence interval (95%CI) to detect odds ratios (OR) of 2.0. The lowest power was observed for the tobacco smoking variable (exposed/unexposed ratio of 1:8).

### Quantitative variables

The age variable was collected continuously (in complete years) and categorized into three age groups (60-69, 70-79 and ≥80 years), taking into account the sample distribution. The other variables analyzed were of a categorical nature.

### Statistical methods

The analyses were performed using the Data Analysis and Statistical Software (Stata14) program. Descriptive analysis was performed by follow-up point based on the calculation of overall influenza vaccination coverage in the last year at each time point of the study and with their respective 95% confidence intervals (CI).

Taking the participants at baseline, and based on the independent variable frequencies, a descriptive analysis was performed of the main characteristics: sociodemographic, behavioral, comorbidities, as well as those related to the use of long-term medications and care at a primary health center in the last year.

The influenza vaccination uptake outcome was analyzed bivariately for the longitudinal study using the chi-square test, which led to the identification of associations between the independent variables and the outcome, considering the proportion of individuals vaccinated over time with 0, 1, 2 and 3 doses of the influenza vaccine.

Unadjusted and adjusted analyses were performed using ordinal logistic regression with OR estimation at a 95% confidence interval. In this model, the ORs represented the cumulative odds of being in a higher vaccination category compared to lower categories.

The multiple analysis was conducted according to a hierarchical conceptual model organized into levels that respected the relationship of distality and proximity between the determinants of vaccination coverage, where the first level meant the most distal, and the last, the most proximal (15).

The variables were grouped into four levels of determination: level 1 (sociodemographic: age group, sex, labor activity and living alone); level 2 (behavioral: tobacco smoking and alcohol consumption in the last week); level 3 (comorbidities: diagnosis of hypertension, diabetes, pulmonary emphysema and asthma); and level 4 (long-term medication use and care at a primary health center in the last year).

The adjustment process was performed within each hierarchical level: all variables at one level were initially included, and those with a p-value>0.200 were sequentially removed until only the variables with a p-value<0.200 remained. Then, the variables from the next level were added, repeating the procedure until the last level was included. In all analyses, a p-value<0.050 was considered statistically significant.

The likelihood ratio was used as a hypothesis test, and the odds proportionality test was used to assess the adequacy of the assumptions of the ordinal logistic regression model.

To minimize biases resulting from loss to follow-up, longitudinal analyses were restricted to the 651 participants present at all three data collection points, thus avoiding missing data in the primary outcome.

No subgroup, interaction or sensitivity analyses were conducted.

## Results 

A total of 651 elderly individuals participating in the Rio Grande EpiRural cohort were included, from the baseline and the two waves of follow-up, representing 63.3% of the initial 2017 sample. Average follow-up was five years (2017-2022).

**Figure 1:**
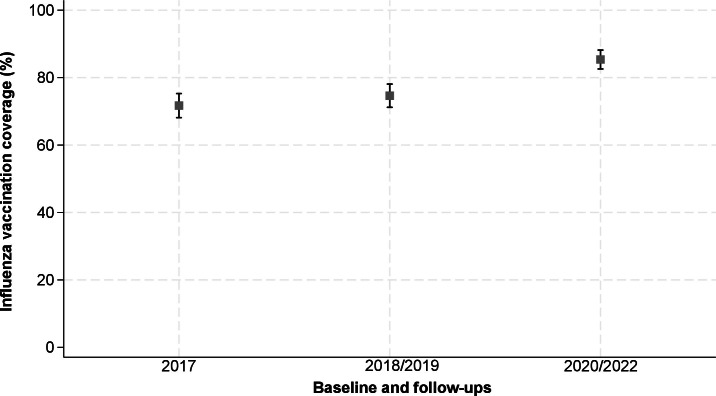
Self-reported influenza vaccination coverage (%), with respective 95% confidence intervals, of the older adults who participated in the EpiRural cohort at baseline in 2017 and in the 2018-2019 and 2020-2022 follow-ups. Rio Grande, 2024 (n=651)

The proportion of participants who reported having been vaccinated against influenza in the previous 12 months progressively increased throughout the cohort follow-up: 71.5% (95%CI 68.0; 75.1%) at baseline, 74.9% (95%CI 71.0; 78.0%) at first follow-up, and 85.7% (95%CI 82.3; 88.0%) at last follow-up. The latter was conducted during the COVID-19 pandemic (Figure 1). These estimates were obtained from follow-up with the same individuals throughout the study, reflecting the evolution of vaccination in the cohort over time.

At baseline, 52.6% of participants were between 60-69 years old and 52.2% were male. Among the variables, 20.1% of older adults reported living alone, 13.7% worked, 10.8% were smokers and 17.4% reported alcohol consumption in the last week. Regarding comorbidities, 57.5% reported diagnosis of hypertension, 14.5% diabetes, 11.6% pulmonary emphysema and 6.6% asthma. 62.1% of the older adults sought care at a primary health care unit in the last year, and 80.6% were taking long-term medication (Table 1).

**Table 1:** Description of the older adults who participated in the EpiRural cohort at baseline in 2017 and in the 2018-2019 and 2020-2022 follow-ups. Rio Grande, 2024 (n=651)

2017 baseline variables	n (%)
**Age group** (years)^a^	
61-69	342 (52.6)
70-79	220 (33.9)
≥80	88 (13.5)
Sex	
Male	340 (52.2)
Female	311 (47.7)
**Lives alone**	
No	520 (79.9)
Yes	131 (20.1)
**Labor activity**	
No	562 (86.3)
Yes	89 (13.7)
**Tobacco smoker**	
No	353 (54.2)
Former smoker	228 (35.0)
Yes	70 (10.8)
**Alcohol consumption in the last week**	
No	538 (82.6)
Yes	113 (17.4)
**Diagnosis of hypert^ensiona^**	
No	276 (42.5)
Yes	374 (57.5)
**Diagnosis of di^abetesa^**	
No	556 (85.5)
Yes	94 (14.5)
**Diagnosis of pulmonary emp^hysemaa^**	
No	549 (88.4)
Yes	72 (11.6)
**Diagnosis of ^asthmaa^**	
No	579 (93.4)
Yes	41 (6.6)
**Care at a primary health center in the las^t^ ^yeara^**	
No	246 (37.9)
Yes	403 (62.1)
**Long-term medication use**	
No	136 (19.4)
Yes	515 (80.6)

^a^Age group (years) (n=650), diagnosis of hypertension (n=650), diagnosis of diabetes (n=650), diagnosis of pulmonary emphysema (n=621), diagnosis of asthma (n=620) and care at a primary health center in the last year (n=649).

In the bivariate analysis of the influenza vaccination uptake outcome (Table 2), significant association was observed between three doses of vaccination coverage in participants who: were not working (67.2%; p-value 0.012); had never smoked (67.7%; p-value<0.001); were former smokers (67.4%; p-value<0.001); sought care at a primary health center in the last year (73.4%; p-value<0.001); and took long-term medication (69.3%; p-value<0.001).

**Table 2:** Bivariate analysis of influenza vaccination uptake and study variables at the end of the second follow-up in older adults from the EpiRural cohort who participated in the baseline in 2017 and in two follow-ups in 2018-2019 and 2020-2022. Rio Grande, 2024 (n=651)

Vaccination uptake	0 dose n (%)	1 dose n (%)	2 doses n (%)	3 doses n (%)	p-value
**Total doses**	65 (10.5)	73 (11.9)	79 (12.9)	397 (64.7)	
Variables					
**Age group** (years)					0.334
60-69	38 (12.1)	44 (14.0)	38 (12.1)	195 (61.9)	
70-79	19 (8.9)	23 (10.8)	26 (12.2)	145 (68.1)	
≥80	8 (9.3)	6 (7.0)	15 (17.4)	57 (66.3)	
Sex					0.065
Male	41 (12.9)	44 (13.8)	37 (11.6)	196 (61.6)	
Female	24 (8.1)	29 (9.8)	42 (14.1)	202 (68.0)	
**Lives alone**					0.091
No	52 (10.6)	50 (10.2)	64 (13.1)	324 (66.1)	
Yes	13 (10.4)	23 (18.4)	15 (12.0)	74 (59.2)	
**Labor activity**					0.012
No	51 (9.6)	59 (11.1)	64 (12.1)	357 (67.2)	
Yes	14 (16.7)	14 (16.7)	15 (17.9)	41 (48.8)	
**Tobacco smoker**					0.002
No	27 (8.2)	35 (10.6)	45 (13.6)	224 (67.7)	
Former smoker	24 (11.2)	24 (11.2)	22 (10.2)	145 (67.4)	
Yes	14 (20.3)	14 (20.3)	12 (17.4)	29 (42.0)	
**Alcohol consumption in the last week**					0.480
No	49 (9.7)	61 (12.1)	64 (12.7)	331 (65.5)	
Yes	16 (14.6)	12 (10.9)	15 (13.6)	67 (60.9)	
**Diagnosis of hypertension**					0.261
No	32 (12.4)	36 (13.9)	34 (13.1)	157 (60.6)	
Yes	33 (9.3)	37 (10.4)	45 (12.7)	240 (67.6)	
**Diagnosis of diabetes**					0.751
No	57 (10.8)	65 (12.3)	66 (12.5)	339 (64.3)	
Yes	8 (9.2)	8 (9.2)	13 (14.9)	58 (66.7)	
**Diagnosis of pulmonary emphysema**					0.086
No	63 (11.6)	66 (12.1)	71(13.0)	344 (63.2)	
Yes	2 (2.8)	7 (9.9)	8(11.3)	54 (76.1)	
**Diagnosis of asthma**					0.468
No	61 (10.7)	71 (12.4)	72 (12.6)	369 (64.4)	
Yes	4 (9.8)	2 (4.9)	7 (17.1)	28 (68.3)	
**Care at a primary health center in the last year**					<0.001
No	34 (14.8)	47 (20.4)	34 (14.8)	115 (50.0)	
Yes	31 (8.1)	26 (6.8)	45 (11.8)	281 (73.4)	
**Long-term medication use**					<0.001
No	23 (18.3)	21 (16.7)	23 (18.3)	59 (46.8)	
Yes	42 (8.6)	52 (10.6)	56 (11.5)	339 (69.3)	

In the adjusted ordinal logistic regression (Table 3), the following remained independently associated with higher odds of vaccination: elderly people who were not working (OR 1.93; 95%CI 1.24; 2.99; p-value 0.004); had never smoked (OR 2.44; 95%CI 1.45; 4.12; p-value<0.001); were former smokers (OR 2.71; 95%CI 1.61; 4.59; p-value<0.001); reported diagnosis of pulmonary emphysema (OR 1.85; 95%CI 1.04; 3.28; p-value 0.036); sought care at a primary health center in the last year (OR 2.55; 95%CI 1.82; 3.63; p-value<0.001); and took long-term medication (OR 1.62; 95%CI 1.06; 2.40; p-value 0.025). Although the sex variable showed a significant effect in the unadjusted analysis, it no longer showed statistical significance when adjusted.

**Table 3:** Odds ratios (OR) and 95% confidence intervals (95%CI) of the unadjusted and adjusted analysis of influenza vaccination uptake, according to study variables, among elderly people who participated in the three follow-ups, in 2017, 2018-2019 and 2020-2022. Rio Grande, 2024 (n=651)

Level^a^	Variables	Unadjusted OR (95%CI)	p-value	Adjusted OR (95%CI)	p-value
	**Age group** (years)		0.134		0.181
	61-69	1.00		1.00	
	70-79	1.34 (0.94; 1.92)		1.35 (0.94; 1.94)	
	≥80	1.30 (0.80; 2.12)		1.24 (0.76; 2.02)	
	Sex		0.038		0.085
	Male	1.00		1.00	
1	Female	1.41 (1.02; 1.95)		1.31 (0.94; 1.82)	
	**Labor activity**		<0.001		0.004
	No	2.04 (1.32; 3.16)		1.93 (1.24; 2.99)	
	Yes	1.00		1.00	
	**Lives alone**		0.140		0.106
	No	1.34 (0.91; 1.97)		1.38 (0.93; 2.04)	
	Yes	1.00		1.00	
	**Tobacco smoker**		<0.001		0.007
	No	2.82 (1.73; 4.58)		2.44 (1.45; 4.12)	
	Former smoker	2.62 (1.57; 4.39)		2.71 (1.61; 4.59)	
	Yes	1.00		1.00	
2	**Alcohol consumption in the last week**		0.227		0.925
	No	1.26 (0.83; 1.90)		1.02 (0.66; 1.58)	
	Yes	1.00		1.00	
	**Diagnosis of hypertension**		0.055		0.289
	No	1.00		1.00	
	Yes	1.37 (0.99; 1.90)		1.20 (0.86; 1.68)	
	**Diagnosis of diabetes**		0.558		0.946
	No	1.00		1.00	
	Yes	1.15 (0.72; 1.83)		0.98 (0.60; 1.60)	
3	**Diagnosis of pulmonary emphysema**		0.021		0.036
	No	1.00		1.00	
	Yes	1.93 (1.10; 3.40)		1.85 (1.04; 3.28)	
	**Diagnosis of asthma**		0.510		0.339
	No	1.00		1.00	
	Yes	1.24 (0.64; 2.43)		0.68 (0.31; 1.49)	
	**Long-term medication use**		<0.001		0.025
	No	1.00		1.00	
	Yes	2.46 (1.68; 1.08)		1.60 (1.06; 2.40)	
4	**Care at a primary health center in the last year**		<0.001		<0.001
	No	1.00		1.00	
	Yes	2.73 (1.96; 3.80)		2.57 (1.82; 3.63)	

^a^Hierarchical levels 1-4, as per the theoretical model used.

The analytical structure of the model was hierarchically organized into four levels, according to the theoretical framework used. The exposure variables kept in the final model underwent correlation analysis to assess for collinearity. The correlations found were classified as very weak and weak.

No correlations were found between variables at the same hierarchical level, which minimized the risk of interference in the estimation of effects. The ordinal logistic regression assumption was assessed using the proportional odds test and achieved a p-value of 0.330.

## Discussion 

A progressive increase in self-reported influenza vaccination coverage was identified among elderly people residing in the rural area of ​​Rio Grande, Rio Grande do Sul, between 2017 and 2022.

Vaccination was more frequent among those who did not work, non-smokers or former smokers, those diagnosed with pulmonary emphysema, those taking long-term medication, and those who sought care at a primary health center in the last year. These findings suggested that linkage to health services and the presence of chronic health conditions were facilitating factors for vaccination, reflecting the role of interactions with the health system in promoting immunization in vulnerable populations.

This study had some limitations. Longitudinal analyses were based only on participants with available information at all three time points investigated within the selected period, which may have introduced attrition bias, since those who remained may have had greater engagement with the health services or better health status. Vaccination was assessed by self-reporting, without documentary validation, and is subject to recall bias. The ordinal logistic regression model used assumed odds proportionality. Although this assumption was checked, local violations could not be ruled out, so the OR should be interpreted as average effects. The choice to select variables with p-value<0.200 in the hierarchical model may have generated instability and selection bias. Given that this is an observational study, the associations described did not allow for causal inference, and the results should be interpreted with caution and generalized only to similar contexts.

In this study, a progressive increase in vaccination coverage was observed, although coverage remained below the annual target of 90% established by the National Immunization Program (10). This highlighted the need for specific strategies to reach the recommended levels of herd protection.

In Brazil, until 2020, the annual target of 90% was being achieved nationally. However, from 2021 onwards, a significant drop was observed (70.9% in 2021, 70.2% in 2022 and 62.3% in 2023) (11,12), a phenomenon partially explained by the updating of the denominator used to calculate vaccination coverage in elderly people, which had not been revised since 2012. Even so, in absolute numbers, about 4 million fewer doses were administered in 2021 compared to 2020 (12), which indicated a real decline in vaccination.

The results of this research corroborated the national scenario of not achieving the target, although they pointed to a rising trend in the rural context of Rio Grande, unlike the national trend. Several hypotheses can explain this divergence. In Rio Grande, during the COVID-19 pandemic, the home vaccination strategy was adopted, which reduced access barriers common in rural areas, such as long distances and transportation difficulties. In addition, from September 2021 onwards, the possibility of co-administering influenza and COVID-19 vaccines may have favored greater coverage (11).

The role of communication was another relevant aspect, since, in rural areas, radio remains an important source of information among older people, which helps to potentially reduce the influence of misinformation disseminated on social networks and messaging applications, more common in urban contexts (16,17). The role of Primary Health Care teams was also highlighted, whose presence in the territory and home visits function as channels for raising awareness and facilitating access to vaccination campaigns.

Distinct scenarios were shown to have occurred among urban elderly populations: increased vaccination coverage in the state of Rio Grande do Sul between 2008 and 2017 (18) and a decrease in the state of Minas Gerais during the pandemic (19). Internationally, results also vary: in Canada, there was an increase in coverage, including during the pandemic (20), while rates remained low in countries such as Italy, Australia and India (21-23). ​​This body of evidence highlighted context heterogeneity and the importance of local analyses, especially in rural areas, which are often underrepresented in research.

In the municipalities of Pelotas and São Paulo, higher coverage was observed among elderly people with advanced age, comorbidities and frequent use of health services, while smokers showed lower vaccination rates (24,25). Similar results were found in other countries, such as Serbia, Hungary, Spain and Mexico, where knowledge about the vaccine, presence of chronic diseases and regular contact with health services proved to be determinants for higher vaccination coverage (26-29). These findings reinforce the results of this study, which identified higher coverage among elderly people with respiratory diseases, long-term medication use and close links with primary care.

In rural China, higher vaccination coverage was identified among elderly people with chronic diseases and in regular contact with health services, highlighting the role of medical follow-up in the decision to vaccinate (7). In Brazil, in riverside communities, geographical access barriers that hindered coverage have been reported, in addition to the importance of the work of local health teams and active search strategies to promote vaccination (8). These findings are consistent with the results of this study, indicating that strengthening primary care and frequent contact with services are key determinants for expanding vaccination in rural areas.

In summary, despite the uncertain scenario of the COVID-19 pandemic, a progressive increase in vaccination coverage was observed, possibly driven by local strategies such as home vaccination and the co-administration of influenza and COVID-19 vaccines. These findings emphasize that regular contact with health services and the presence of clinical conditions requiring continuous monitoring are valuable opportunities for promoting vaccination. Although reflecting the specific reality of the rural elderly population of Rio Grande, the results provide support for guiding interventions in similar rural contexts, in Brazil and in other countries that have comparable challenges to access to healthcare.

## Data Availability

The database and analysis codes used in the research are not available online. To obtain them, please contact the corresponding author.
