# Supplementary material for: Monitoring influenza vaccination coverage among older adults: a rural cohort study, Rio Grande, 2017-2022
Source: Epidemiol Serv Saude. 2026 Mar 23;35:e20250561. doi: 10.1590/S2237-96222026v35e20250561.en (PMC13012720; doi:10.1590/S2237-96222026v35e20250561.en)
Supplement: Translation

# Translation: Monitoramento da cobertura vacinal contra influenza em pessoas idosas: estudo de coorte rural, Rio Grande, 2017-2022

**DOI:** 10.1590/S2237-96222026v35e20250561.pt

**Authors:** Ana Claudia Klein de Almeida de Chaves, Rodrigo Dalke Meucci

**Affiliations:** 1 Universidade Federal do Rio Grande, Saúde Pública, Rio Grande, RS, Brasil; 2 Universidade Federal do Rio Grande, Faculdade de Medicina, Programa de Pós Graduação em Saúde Pública, Rio Grande, RS, Brasil

**Keywords:** Cobertura Vacinal, Vacinas Contra Influenza, Pessoa Idosa, Zona Rural, Saúde da População Rural

## Abstract

**Objetivos:**

Estimar a cobertura vacinal contra influenza em pessoas idosas da coorte rural de Rio Grande, no Rio Grande do Sul, a partir de dados da linha de base em 2017 e dos acompanhamentos em 2018-2019 e em 2020-2022, e identificar os fatores associados à trajetória de vacinação nesses períodos.

**Métodos:**

Estudo de coorte, parte do projeto EpiRural Rio Grande, realizado em três momentos: linha de base em 2017 e acompanhamentos em 2018-2019 e em 2020-2022. Foram incluídos 651 participantes com 60 anos ou mais presentes em todas as ondas. A informação sobre vacinação foi autorrelatada. Foram estimadas proporções de cobertura vacinal (%) em cada ponto de seguimento. A análise de fatores associados à vacinação utilizou regressão logística ordinal hierárquica para estimar razões de chances (*odds ratio*, OR) com intervalos de confiança de 95% (IC95%).

**Resultados:**

A cobertura vacinal contra influenzaaumentou de 71,5% na linha de base para 85,7% em 2020-2022. Na análise ajustada, a maior chance de vacinação esteve associada a não estar em atividade laboral (OR 1,93; IC95% 1,24; 2,99), não fumar (OR 2,44; IC95% 1,45; 4,12), ser ex-fumante (OR 2,71; IC95% 1,61; 4,59), diagnóstico de enfisema pulmonar (OR 1,85; IC95% 1,04; 3,28), utilizar medicamentos contínuos (OR 1,62; IC95% 1,06; 2,40) e buscar atendimento em unidade básica de saúde no último ano (OR 2,55; IC95% 1,82; 3,63).

**Conclusão:**

A cobertura vacinal contra influenza aumentou ao longo do tempo, embora abaixo da meta nacional, e foi maior entre participantes com vínculo aos serviços de saúde e com condições crônicas.

**Aspectos éticos**

Esta pesquisa respeitou os princípios éticos, obtendo os seguintes dados de aprovação:

Comitê de ética em pesquisa: Universidade Federal do Rio Grande

Número do parecer: 15/4/2018

Data de aprovação: 2/8/2018

Certificado de apresentação de apreciação ética: 70294317.0.0000.5324

Registro do consentimento livre e esclarecido: Obtido de todos os participantes antes da coleta.

## Introdução 

A partir de 1955, a população mundial com 60 anos ou mais sextuplicou e estima-se que esse grupo se torne majoritário nas próximas décadas (1). No Brasil, pessoas idosas são definidas como aquelas com 60 anos ou mais (2). Entre os Censos Demográficos 2010 e 2022, a população idosa cresceu 56%, e o Rio Grande do Sul foi o estado com a maior proporção, 20% (3).

O envelhecimento populacional está associado ao aumento das condições crônicas de saúde e à maior vulnerabilidade a doenças infecciosas, como as respiratórias. Estima-se que, a cada ano, ocorram cerca de 1 bilhão de casos de influenza no mundo, dos quais 3 a 5 milhões evoluem para formas graves, resultando em até 650 mil mortes (4). No Brasil, em 2024, pessoas idosas representaram 40% das hospitalizações por síndrome respiratória aguda grave, causada por influenza, com letalidade de 66% (5).

A vacinação contra influenza é uma das principais estratégias de saúde pública para reduzir casos graves, hospitalizações e óbitos, especialmente em pessoas idosas. No Brasil, a vacinação é ofertada pelo Sistema Único de Saúde anualmente desde 1999, com pessoas idosas no grupo prioritário (6).

Entretanto, a cobertura vacinal contra influenza em pessoas idosas rurais não tem sido explorada nas pesquisas nacionais e internacionais, assim poucos estudos abordam essa população (7,8). Estima-se que pessoas idosas residentes em zonas rurais enfrentem maiores dificuldades de acesso a serviços de saúde, devido a distâncias, menor renda e foco em atendimento curativo, o que pode aumentar a vulnerabilidade e prejudicar a qualidade de vida (9).

Desde 2017, a meta nacional anual de cobertura vacinal contra influenza é de 90% (10). Embora em anos anteriores a cobertura tenha alcançado essa meta, sua avaliação em diferentes momentos é essencial para compreender a evolução da vacinação entre pessoas idosas, sobretudo em zonas rurais, onde existem barreiras de acesso aos serviços de saúde. O período de 2020-2022, marcado pela pandemia da covid-19, representa um contexto singular, com possíveis impactos tanto na procura quanto na oferta da vacina.

A partir de 2021, as coberturas vacinais passaram a ficar abaixo da meta nacional (11), mesmo considerando a atualização do denominador utilizado para o cálculo da cobertura vacinal em pessoas idosas. Ainda assim, o número total de doses administradas foi 4 milhões a menos do que em 2020. Em 2022, em 2023 e em 2024, a cobertura também permaneceu abaixo do esperado (12). Isso reforça a importância de estudos que investiguem a evolução da cobertura em períodos anteriores e identifiquem fatores associados à vacinação, o que contribui para o monitoramento de tendências e para o planejamento de estratégias de saúde pública.

Os objetivos deste estudo foram estimar a cobertura vacinal contra influenza em pessoas idosas da coorte rural de Rio Grande, no Rio Grande do Sul, a partir de dados da linha de base em 2017 e dos acompanhamentos em 2018-2019 e em 2020-2022, e identificar os fatores associados à trajetória de vacinação nestes períodos.

## Métodos 

### Delineamento 

Trata-se de estudo recorte da pesquisa “EpiRural Rio Grande: coorte de pessoas idosas da zona rural no município de Rio Grande, no Rio Grande do Sul”, cujo delineamento metodológico foi descrito previamente (13). O objetivo geral da coorte é descrever e monitorar os padrões de morbimortalidade e de utilização de serviços de saúde entre pessoas idosas residentes na zona rural de Rio Grande.

O estudo constitui uma coorte prospectiva com o acompanhamento dessa população em três momentos distintos. A linha de base foi realizada em 2017, com duas ondas de acompanhamento: a primeira entre 2018 e 2019 e a segunda entre 2020 e 2022 – esta última foi dividida em dois períodos, devido a consequências da pandemia da covid-19, (3/11/2020-26/11/2020 e 4/10/2021-19/1/2022). Para as análises, foram considerados os 651 participantes presentes nos três momentos.

### Contexto 

Este estudo foi desenvolvido na zona rural de Rio Grande, localizado no Rio Grande do Sul, extremo sul do Brasil. 

À época do estudo, o município possuía aproximadamente 191 mil habitantes e, em 2022, 8.383 pessoas residiam em zona rural, o que representou 4,4% da população total (14). A zona rural do município apresentou grande extensão territorial e baixa densidade populacional, o que dificultou o acesso a serviços.

A linha de base ocorreu em 2017 e os acompanhamentos em 2018-2019 e em 2020-2022, o que permitiu avaliar a vacinação em diferentes momentos, inclusive no período da pandemia da covid-19.

### Participantes 

Foram incluídas no estudo pessoas com 60 anos ou mais residentes na zona rural de Rio Grande, conforme definição do Estatuto do Idoso (2). Foram excluídas aquelas hospitalizadas ou institucionalizadas no período da aplicação dos questionários.

A amostra foi de base populacional e foi obtida a partir de inquérito domiciliar realizado na zona rural do município. O acompanhamento dos participantes se deu por meio de visitas domiciliares em três momentos de coleta (2017, 2018-2019 e 2020-2022). 

Para as análises longitudinais, foram considerados todos os indivíduos presentes nas três etapas da pesquisa, o que totalizou 651 participantes.

### Variáveis 

O desfecho para estimar a cobertura vacinal contra influenza nos diferentes tempos se concentrou na resposta à pergunta: “O(a) Senhor(a) tomou alguma dose desta vacina desde <mês> do ano passado para cá?”. As opções de resposta foram “sim” e “não”. Essa pergunta possibilitou avaliar a cobertura vacinal anual, conforme recomendado pelo Ministério da Saúde (11). A pergunta se repetiu de forma igual nos três momentos. 

Para avaliar os fatores associados à trajetória de vacinação contra influenza, foi construída uma variável ordinal, derivada da soma das respostas afirmativas à pergunta sobre a vacinação contra influenza nos três períodos avaliados (linha de base, primeira onda de seguimento e segunda onda de seguimento). A variável resultante apresentou quatro categorias: 0, 1, 2 e 3 doses de vacina recebidas ao longo do tempo, o que foi interpretado como indicador da trajetória de vacinação contra influenza durante o período do estudo.

As variáveis de exposição foram agrupadas em sociodemográficas (faixa etária, sexo, atividade laboral e morar sozinho). As características comportamentais mensuradas incluíram tabagismo e consumo de álcool na última semana. A pergunta referente ao tabagismo apresentou três opções de resposta: nunca fumou, fumante e ex-fumante. 

As variáveis relacionadas às condições de saúde incluíram o diagnóstico de hipertensão, diabetes, enfisema pulmonar e asma. Além dessas, foram consideradas as variáveis referentes ao uso de medicamentos contínuos e ao atendimento em unidade básica de saúde no último ano.

Essas variáveis foram consideradas potenciais fatores associados à vacinação e incluídas no modelo hierárquico como possíveis preditores e confundidores.

### Fonte de dados e mensuração 

Os dados utilizados neste estudo foram provenientes da coorte EpiRural. Em todos os momentos de coleta (linha de base, primeiro e segundo acompanhamentos), foi utilizado um questionário padronizado e pré-codificado, aplicado por entrevistadoras treinadas em visitas domiciliares, com registro em tablets por meio do programa Research Electronic Data Capture.

As informações sobre vacinação, características sociodemográficas, características comportamentais, condições de saúde e uso de serviços de saúde foram obtidas por autorrelato do participante e, quando não possível, por seu cuidador. 

Os mesmos instrumentos e procedimentos de coleta foram adotados em todos os períodos, o que garantiu a comparabilidade entre os diferentes momentos do estudo.

### Viés 

Para minimizar vieses de informação, utilizou-se questionário padronizado, aplicado por entrevistadoras treinadas, com a mesma pergunta sobre vacinação repetida nos três momentos de coleta. 

Reconheceu-se a possibilidade de viés de atrito devido às perdas de seguimento. Para reduzir esse efeito, a análise foi restrita aos 651 participantes presentes em todas as ondas. Potenciais confundidores foram controlados por meio de regressão multivariada hierárquica.

### Tamanho do estudo 

Na linha de base do estudo, realizada em 2017, foram amostrados 2.218 domicílios, aproximadamente 80% do total da zona rural de Rio Grande. A seleção foi feita por amostragem aleatória sistemática em todos os setores censitários, com inclusão de quatro a cada cinco domicílios consecutivos. Nesses domicílios, identificaram-se 1.130 pessoas com 60 anos ou mais, das quais 1.029 participaram da pesquisa, o que constituiu a amostra inicial da coorte (13).

No primeiro seguimento, em 2018-2019, 862 pessoas idosas foram reentrevistadas (taxa de acompanhamento: 83,8%). No segundo seguimento, em 2020-2022, 651 participaram (63,3% da amostra original). Para as análises longitudinais, foram incluídos exclusivamente os 651 indivíduos presentes em todos os três momentos, a fim de assegurar a comparabilidade entre os períodos.

Foi realizada a análise de poder com a consideração da amostra final de 651 indivíduos. O tamanho amostral conferiu poder estatístico mínimo de 80%, com intervalo de confiança de 95% (IC95%) para detectar razões de chances (*odds ratio*, OR) de 2,0. O menor poder foi observado para a variável tabagismo (proporção expostos/não expostos de 1:8).

### Variáveis quantitativas

A variável idade foi coletada de forma contínua (em anos completos) e categorizada em faixa etária com três grupos (60-69, 70-79 e ≥80 anos), com a consideração da distribuição da amostra. As demais variáveis analisadas apresentaram natureza categórica.

### Métodos estatísticos

As análises foram realizadas no programa estatístico Data Analysis and Statistical Software (Stata14). Foi realizada análise descritiva por ponto de seguimento a partir do cálculo das coberturas gerais de vacinação contra influenza no último ano em cada tempo do estudo e com seus respectivos IC95%. 

Dos participantes na linha de base, com a apresentação das frequências das variáveis independentes, foi realizada análise descritiva das principais características: sociodemográficas, comportamentais, de comorbidades e referentes ao uso de medicamentos contínuos e ao atendimento em unidade básica de saúde no último ano. 

O desfecho trajetória de vacinação contra influenza foi analisado de forma bivariada para o estudo longitudinal com a utilização do teste qui-quadrado, o que levou à identificação de associações entre as variáveis independentes e o desfecho com a consideração da a proporção de indivíduos vacinados ao longo do tempo com 0, 1, 2 e 3 doses da vacina contra influenza.

Foram realizadas análises brutas e ajustadas por meio de regressão logística ordinal com a estimativa de OR com IC95%. Nesse modelo, as OR representaram a chance acumulada de se estar em uma categoria mais elevada de vacinação em comparação às inferiores.

A análise múltipla foi conduzida segundo um modelo hierárquico conceitual organizado em níveis que respeitaram a relação de distalidade e de proximidade entre os determinantes da cobertura vacinal, em que o primeiro nível significava o mais distal, e o último, o mais proximal (15).

As variáveis foram agrupadas em quatro níveis de determinação: nível 1 (sociodemográficas: faixa etária, sexo, atividade laboral e morar sozinho); nível 2 (comportamentais: tabagismo e consumo de álcool na última semana); nível 3 (comorbidades: diagnóstico de hipertensão, diabetes, enfisema pulmonar e asma); e nível 4 (uso de medicamento contínuo e atendimento em unidade básica de saúde no último ano).

O processo de ajuste foi realizado dentro de cada nível hierárquico: todas as variáveis de um nível foram inicialmente incluídas, e aquelas com p-valor>0,200 foram retiradas sequencialmente, até permanecerem apenas as variáveis com p-valor<0,200. Em seguida, foram acrescentadas as variáveis do nível seguinte, repetindo-se o procedimento até a inclusão do último nível. Em todas as análises, considerou-se p-valor<0,050 como estatisticamente significativo.

Utilizou-se a razão de verossimilhanças como teste de hipóteses e, para avaliar a adequação dos pressupostos do modelo de regressão logística ordinal, foi usado o teste de proporcionalidade das chances.

Para minimizar vieses decorrentes de perdas de seguimento, as análises longitudinais foram restritas aos 651 participantes presentes nos três momentos de coleta, o que evitou dados faltantes no desfecho principal. 

Não foram conduzidas análises de subgrupos, de interações ou de sensibilidade.

## Resultados 

Foram incluídas 651 pessoas idosas participantes da coorte EpiRural de Rio Grande da linha de base e das duas ondas de acompanhamentos, o que representou 63,3% da amostra inicial de 2017. O seguimento médio foi de cinco anos (2017-2022).

A proporção de participantes que referiram terem sido vacinados contra influenza nos 12 meses anteriores aumentou progressivamente ao longo do seguimento da coorte: 71,5% (IC95% 68,0; 75,1%) no momento da linha de base, 74,9% (IC95% 71,0; 78,0%) no primeiro acompanhamento e 85,7% (IC95% 82,3; 88,0%) no último acompanhamento, realizado durante a pandemia de covid-19 (Figura 1). Essas estimativas foram obtidas a partir do seguimento dos mesmos indivíduos ao longo do estudo, o que refletiu a evolução da vacinação na coorte ao longo do tempo.

**Figura 1:** Cobertura (%) autorrelatada de vacinação contra influenza, com os respectivos intervalos de confiança de 95%, de pessoas idosas que participaram da coorte EpiRural na linha de base em 2017 e nos acompanhamentos em 2018-2019 e em 2020-2022. Rio Grande, 2024 (n=651)

Na linha de base, 52,6% dos participantes tinha entre 60-69 anos e 52,2% era do sexo masculino. Dentro das variáveis, 20,1% das pessoas idosas referiram morar sozinhas, 13,7% estavam em atividade laboral, 10,8% eram fumantes e 17,4% relataram consumo de álcool na última semana. Das comorbidades, 57,5% relataram diagnóstico de hipertensão, 14,5% de diabetes, 11,6% de enfisema pulmonar e 6,6% de asma. Procuraram atendimento na unidade básica de saúde no último ano 62,1% das pessoas idosas, e 80,6% usavam medicação contínua (Tabela 1).

**Tabela 1:** Descrição das pessoas idosas da coorte EpiRural que participaram da linha de base em 2017 e de dois acompanhamentos nos períodos 2018-2019 e 2020-2022, segundo as variáveis do estudo na linha de base. Rio Grande, 2024 (n=651)

Variáveis da linha de base 2017	n (%)
**Faixa etária** (anos)^a^	
61-69	342 (52,6)
70-79	220 (33,9)
≥80	88 (13,5)
Sexo	
Masculino	340 (52,2)
Feminino	311 (47,7)
**Morar sozinho**	
Não	520 (79,9)
Sim	131 (20,1)
**Atividade laboral**	
Não	562 (86,3)
Sim	89 (13,7)
Tabagismo	
Não	353 (54,2)
Ex-fumante	228 (35,0)
Sim	70 (10,8)
**Consumo de álcool na última semana**	
Não	538 (82,6)
Sim	113 (17,4)
**Diagnóstico de hipertensão^a^**	
Não	276 (42,5)
Sim	374 (57,5)
**Diagnóstico de diabetes^a^**	
Não	556 (85,5)
Sim	94 (14,5)
**Diagnóstico de enfisema pulmonar^a^**	
Não	549 (88,4)
Sim	72 (11,6)
**Diagnóstico de asma^a^**	
Não	579 (93,4)
Sim	41 (6,6)
**Atendimento em unidade básica de saúde no último ano^a^**	
Não	246 (37,9)
Sim	403 (62,1)
**Uso de medicação contínua**	
Não	136 (19,4)
Sim	515 (80,6)

^a^Faixa etária (anos) (n=650), diagnóstico de hipertensão (n=650), diagnóstico de diabetes (n=650), diagnóstico de enfisema pulmonar (n=621), diagnóstico de asma (n=620) e atendimento em unidade básica de saúde no último ano (n=649).

Na análise bivariada do desfecho trajetória de vacinação contra influenza (Tabela 2), observou-se associação significativa da cobertura vacinal de três doses nos participantes que: estavam sem atividade laboral (67,2%; p-valor 0,012); nunca fumaram (67,7%; p-valor<0,001); eram ex-fumantes (67,4%; p-valor<0,001); procuraram atendimento em unidade básica de saúde no último ano (73,4%; p-valor<0,001); e usavam medicação contínua (69,3%; p-valor<0,001). 

**Tabela 2:** Análise bivariada da trajetória de vacinação contra influenza e das variáveis do estudo ao final do segundo acompanhamento em pessoas idosas da coorte EpiRural que participaram da linha de base em 2017 e de dois acompanhamentos nos períodos 2018-2019 e 2020-2022. Rio Grande, 2024 (n=651)

Trajetória de vacinação	0 dose n (%)	1 dose n (%)	2 doses n (%)	3 doses n (%)	p-valor
**Total de doses**	65 (10,5)	73 (11,9)	79 (12,9)	397 (64,7)	
Variáveis					
**Faixa etária** (anos)					0,334
60-69	38 (12,1)	44 (14,0)	38 (12,1)	195 (61,9)	
70-79	19 (8,9)	23 (10,8)	26 (12,2)	145 (68,1)	
≥80	8 (9,3)	6 (7,0)	15 (17,4)	57 (66,3)	
Sexo					0,065
Masculino	41 (12,9)	44 (13,8)	37 (11,6)	196 (61,6)	
Feminino	24 (8,1)	29 (9,8)	42 (14,1)	202 (68,0)	
**Morar sozinho**					0,091
Não	52 (10,6)	50 (10,2)	64 (13,1)	324 (66,1)	
Sim	13 (10,4)	23 (18,4)	15 (12,0)	74 (59,2)	
**Atividade laboral**					0,012
Não	51 (9,6)	59 (11,1)	64 (12,1)	357 (67,2)	
Sim	14 (16,7)	14 (16,7)	15 (17,9)	41 (48,8)	
Tabagismo					0,002
Não	27 (8,2)	35 (10,6)	45 (13,6)	224 (67,7)	
Ex-fumante	24 (11,2)	24 (11,2)	22 (10,2)	145 (67,4)	
Sim	14 (20,3)	14 (20,3)	12 (17,4)	29 (42,0)	
**Consumo de álcool na última semana**					0,480
Não	49 (9,7)	61 (12,1)	64 (12,7)	331 (65,5)	
Sim	16 (14,6)	12 (10,9)	15 (13,6)	67 (60,9)	
**Diagnóstico de hipertensão**					0,261
Não	32 (12,4)	36 (13,9)	34 (13,1)	157 (60,6)	
Sim	33 (9,3)	37 (10,4)	45 (12,7)	240 (67,6)	
**Diagnóstico de diabetes**					0,751
Não	57 (10,8)	65 (12,3)	66 (12,5)	339 (64,3)	
Sim	8 (9,2)	8 (9,2)	13 (14,9)	58 (66,7)	
**Diagnóstico de enfisema pulmonar**					0,086
Não	63 (11,6)	66 (12,1)	71(13,0)	344 (63,2)	
Sim	2 (2,8)	7 (9,9)	8(11,3)	54 (76,1)	
**Diagnóstico de asma**					0,468
Não	61 (10,7)	71 (12,4)	72 (12,6)	369 (64,4)	
Sim	4 (9,8)	2 (4,9)	7 (17,1)	28 (68,3)	
**Atendimento em unidade básica de saúde no último ano**					<0,001
Não	34 (14,8)	47 (20,4)	34 (14,8)	115 (50,0)	
Sim	31 (8,1)	26 (6,8)	45 (11,8)	281 (73,4)	
**Uso de medicação contínua**					<0,001
Não	23 (18,3)	21 (16,7)	23 (18,3)	59 (46,8)	
Sim	42 (8,6)	52 (10,6)	56 (11,5)	339 (69,3)	

Na regressão logística ordinal ajustada (Tabela 3), permaneceram associadas, de forma independente à maior chance de vacinação, as pessoas idosas que: estavam sem atividade laboral (OR 1,93; IC95% 1,24; 2,99; p-valor 0,004); nunca fumaram (OR 2,44; IC95% 1,45; 4,12; p-valor<0,001); eram ex-fumantes (OR 2,71; IC95% 1,61; 4,59; p-valor<0,001); relataram diagnóstico de enfisema pulmonar (OR 1,85; IC95% 1,04; 3,28; p-valor 0,036); procuraram atendimento em unidade básica de saúde no último ano (OR 2,55; IC95% 1,82; 3,63; p-valor<0,001); e usavam medicamento contínuo (OR 1,62; IC95% 1,06; 2,40; p-valor 0,025). Embora a variável sexo tenha mostrado efeito significativo na análise bruta, ao ser ajustada não apresentou mais significância estatística.

**Tabela 3:** Razão de chances (*odds ratio*, OR) e intervalo de confiança de 95% (IC95%) da análise bruta e ajustada da trajetória de vacinação contra influenza, segundo variáveis do estudo, com pessoas idosas que participaram dos três acompanhamentos, em 2017, em 2018-2019 e em 2020-2022. Rio Grande, 2024 (n=651)

Nível^a^	Variáveis	OR bruta (IC95%)	p-valor	OR ajustada (IC95%)	p-valor
	Faixa etária (anos)		0,134		0,181
	61-69	1,00		1,00	
	70-79	1,34 (0,94; 1,92)		1,35 (0,94; 1,94)	
	≥80	1,30 (0,80; 2,12)		1,24 (0,76; 2,02)	
	Sexo		0,038		0,085
	Masculino	1,00		1,00	
1	Feminino	1,41 (1,02; 1,95)		1,31 (0,94; 1,82)	
	Atividade laboral		<0,001		0,004
	Não	2,04 (1,32; 3,16)		1,93 (1,24; 2,99)	
	Sim	1,00		1,00	
	Morar sozinho		0,140		0,106
	Não	1,34 (0,91; 1,97)		1,38 (0,93; 2,04)	
	Sim	1,00		1,00	
	Tabagismo		<0,001		0,007
	Não	2,82 (1,73; 4,58)		2,44 (1,45; 4,12)	
	Ex-fumante	2,62 (1,57; 4,39)		2,71 (1,61; 4,59)	
	Sim	1,00		1,00	
2	Consumo de álcool na última semana		0,227		0,925
	Não	1,26 (0,83; 1,90)		1,02 (0,66; 1,58)	
	Sim	1,00		1,00	
	Diagnóstico de hipertensão		0,055		0,289
	Não	1,00		1,00	
	Sim	1,37 (0,99; 1,90)		1,20 (0,86; 1,68)	
	Diagnóstico de diabetes		0,558		0,946
	Não	1,00		1,00	
	Sim	1,15 (0,72; 1,83)		0,98 (0,60; 1,60)	
3	Diagnóstico de enfisema pulmonar		0,021		0,036
	Não	1,00		1,00	
	Sim	1,93 (1,10; 3,40)		1,85 (1,04; 3,28)	
	Diagnóstico de asma		0,510		0,339
	Não	1,00		1,00	
	Sim	1,24 (0,64; 2,43)		0,68 (0,31; 1,49)	
	Uso de medicação contínua		<0,001		0,025
	Não	1,00		1,00	
	Sim	2,46 (1,68; 1,08)		1,60 (1,06; 2,40)	
4	Atendimento em unidade básica de saúde no último ano		<0,001		<0,001
	Não	1,00		1,00	
	Sim	2,73 (1,96; 3,80)		2,57 (1,82; 3,63)	

^a^Níveis hierárquicos 1-4, conforme modelo teórico adotado.

A estrutura analítica do modelo foi hierarquizada em quatro níveis, conforme o referencial teórico adotado. As variáveis de exposição mantidas no modelo final foram submetidas à análise de correlação para avaliação da colinearidade. As correlações observadas foram classificadas entre muito fracas e fracas. 

Eventuais correlações não ocorreram entre variáveis de um mesmo nível hierárquico, o que minimizou o risco de interferência na estimação dos efeitos. O pressuposto de proporcionalidade de chances da regressão logística ordinal foi testado e atendido com p-valor 0,330. 

## Discussão 

Identificou-se aumento progressivo na cobertura autorrelatada de vacinação contra influenza entre as pessoas idosas residentes na zona rural de Rio Grande, no Rio Grande do Sul, entre 2017 e 2022. 

A vacinação foi mais frequente entre aquelas que estavam sem atividade laboral, não fumavam ou eram ex-fumantes, com diagnóstico de enfisema pulmonar, que utilizavam medicação contínua e que procuraram atendimento em unidade básica de saúde no último ano. Esses achados sugeriram que o vínculo com os serviços de saúde e a presença de condições crônicas de saúde foram fatores facilitadores para a realização da vacina, o que refletiu o papel das interações com o sistema de saúde na promoção da imunização em populações vulneráveis.

Este estudo apresentou algumas limitações. As análises longitudinais basearam-se apenas nos participantes com informação disponível nos três momentos investigados no período recortado, o que pôde introduzir viés de atrito, já que aqueles que permaneceram puderam ter maior engajamento com os serviços, ou melhor estado de saúde. A vacinação foi aferida por autorreferência, sem validação documental, sujeita a erro de recordatório. O modelo de regressão logística ordinal utilizado pressupôs proporcionalidade das chances. Embora esse pressuposto tenha sido avaliado, violações locais não puderam ser descartadas, de modo que as OR devessem ser interpretadas como efeitos médios. A opção por seleçãomantendo variáveis com p-valor <0,200 no modelo hierárquico pôde gerar instabilidade e viés de seleção de variáveis. Por se tratar de estudo observacional, as associações descritas não permitiram inferência causal, devendo os resultados ser interpretados com cautela e generalizados apenas a contextos semelhantes.

Neste estudo, observou-se aumento progressivo da cobertura vacinal, embora a cobertura tenha permanecido abaixo da meta anual de 90% estabelecida pelo Programa Nacional de Imunizações (10). Isso reforçou a necessidade de estratégias específicas para atingir os níveis recomendados de proteção coletiva.

No Brasil, até 2020, a meta anual de 90% vinha sendo alcançada em nível nacional. Contudo, a partir de 2021, observou-se queda expressiva (70,9% em 2021, 70,2% em 2022 e 62,3% em 2023) (11,12), fenômeno parcialmente explicado pela atualização do denominador utilizado para o cálculo da cobertura vacinal em pessoas idosas, que não era revisto desde 2012. Ainda assim, em números absolutos, cerca de 4 milhões de doses a menos foram aplicadas em 2021 quando comparado a 2020 (12), o que indicou declínio real na vacinação. 

Os resultados desta pesquisa corroboraram o cenário nacional de não alcance da meta, embora tenham apontado para uma tendência de crescimento no contexto rural de Rio Grande, diferentemente da tendência nacional. Algumas hipóteses podem explicar essa divergência. Em Rio Grande, durante a pandemia de covid-19, adotou-se a estratégia de vacinação domiciliar, o que reduziu barreiras de acesso comuns em zonas rurais, como longas distâncias e dificuldades de transporte. Além disso, a partir de setembro de 2021, a possibilidade de coadministração das vacinas contra influenza e contra covid-19 pôde ter favorecido maior cobertura (11). 

O papel da comunicação foi outro aspecto relevante, visto que, em zonas rurais, o rádio permanece como importante fonte de informação entre pessoas idosas, o que ajuda a reduzir potencialmente a influência da desinformação disseminada em redes sociais e em aplicativos de mensagens, mais comuns em contextos urbanos (16,17). Destacou-se também o papel das equipes de Atenção Primária à Saúde, cuja presença no território e visitas domiciliares funcionam como canais de sensibilização e de facilitação do acesso às campanhas de vacinação.

Foram mostrados cenários distintos em populações de pessoas idosas urbanas: aumento das coberturas vacinais no Rio Grande do Sul entre 2008 e 2017 (18) e queda em Minas Gerais durante a pandemia (19). Em âmbito internacional, resultados também variam: no Canadá, houve aumento nas coberturas, inclusive durante a pandemia (20), enquanto as taxas permaneceram baixas em países como Itália, Austrália e Índia (21-23). Esse conjunto de evidências reforçou a heterogeneidade dos contextos e a importância de análises locais, especialmente em zonas rurais, frequentemente sub-representadas nas pesquisas. 

Em Pelotas e no município de São Paulo, foi constatada maior cobertura entre pessoas idosas com idade avançada, comorbidades e uso frequente dos serviços de saúde, enquanto fumantes apresentaram menor vacinação (24,25). Resultados semelhantes foram encontrados em outros países, como Sérvia, Hungria, Espanha e México, onde o conhecimento sobre a vacina, a presença de doenças crônicas e o contato regular com serviços de saúde se mostraram determinantes para maior cobertura vacinal (26-29). Esses achados reforçam os resultados deste estudo, que identificou maior cobertura entre pessoas idosas com doenças respiratórias, uso de medicação contínua e vínculos estreitos com a atenção primária.

Na zona rural da China, identificou-se maior cobertura vacinal entre pessoas idosas com doenças crônicas e em contato regular com serviços de saúde, o que destaca o papel do acompanhamento médico na decisão de se vacinar (7). No Brasil, em comunidades ribeirinhas, foram relatadas barreiras de acesso geográfico que dificultaram a cobertura, além da importância da atuação de equipes de saúde locais e das estratégias de busca ativa para favorecer a vacinação (8). Tais achados dialogaram com os resultados deste estudo, o que indica que o fortalecimento da atenção primária e o contato frequente com os serviços são determinantes centrais para ampliar a vacinação em zonas rurais.

Em síntese, apesar do cenário incerto da pandemia de covid-19, observou-se aumento progressivo da cobertura vacinal, possivelmente impulsionado por estratégias locais, como a vacinação domiciliar e a coadministração de vacinas contra influenza e contra covid-19. Esses achados reforçam que o contato regular com os serviços de saúde e a presença de condições clínicas que demandam acompanhamento contínuo constituem oportunidades valiosas para promover a vacinação. Embora reflitam realidade específica da população idosa rural de Rio Grande, os resultados ofereceram subsídios para orientar intervenções em contextos rurais semelhantes, no Brasil e em outros países com desafios comparáveis de acesso à saúde.

## Data Availability

O banco de dados e os códigos de análise utilizados na pesquisa não estão disponíveis em site. Para obtê-los, é necessário entrar em contato com a autora correspondente.
